# Transition metal-catalysed A-ring C–H activations and C(sp^2^)–C(sp^2^) couplings in the 13α-oestrone series and *in vitro* evaluation of antiproliferative properties

**DOI:** 10.1080/14756366.2021.1900165

**Published:** 2021-03-26

**Authors:** Péter Traj, Ali Hazhmat Abdolkhaliq, Anett Németh, Sámuel Trisztán Dajcs, Ferenc Tömösi, Tea Lanisnik-Rizner, István Zupkó, Erzsébet Mernyák

**Affiliations:** a Department of Organic Chemistry, University of Szeged, Szeged, Hungary; b Department of Pharmacodynamics and Biopharmacy, University of Szeged, Szeged, Hungary; c Department of Medicinal Chemistry, University of Szeged, Szeged, Hungary; d Institute of Biochemistry, Faculty of Medicine, University of Ljubljana, Ljubljana, Slovenia

**Keywords:** 13α-oestrone, C‒H activation, Suzuki–Miyaura coupling, human reproductive cell lines, antiproliferative action

## Abstract

Facile syntheses of 3-*O*-carbamoyl, -sulfamoyl, or -pivaloyl derivatives of 13α-oestrone and its 17-deoxy counterpart have been carried out. Microwave-induced, Ni-catalysed Suzuki–Miyaura couplings of the newly synthesised phenol esters with phenylboronic acid afforded 3-deoxy-3-phenyl-13α-oestrone derivatives. The carbamate and pivalate esters proved to be suitable for regioselective arylations. 2-(4-Substituted) phenyl derivatives were synthesised via Pd-catalysed, microwave-assisted C–H activation reactions. An efficient, one-pot, tandem methodology was elaborated for the introduction of the carbamoyl or pivaloyl group followed by regioselective C-2-arylation and subsequent removal of the directing group. The antiproliferative properties of the novel 13α-oestrone derivatives were evaluated *in vitro* on five human adherent cancer cell lines of gynaecological origin. 3-Sulfamate derivatives displayed substantial cell growth inhibitory potential against certain cell lines. The newly identified antiproliferative compounds having hormonally inactive core might be promising candidates for the design of more active anticancer agents.

## Introduction

**Scheme 1.**
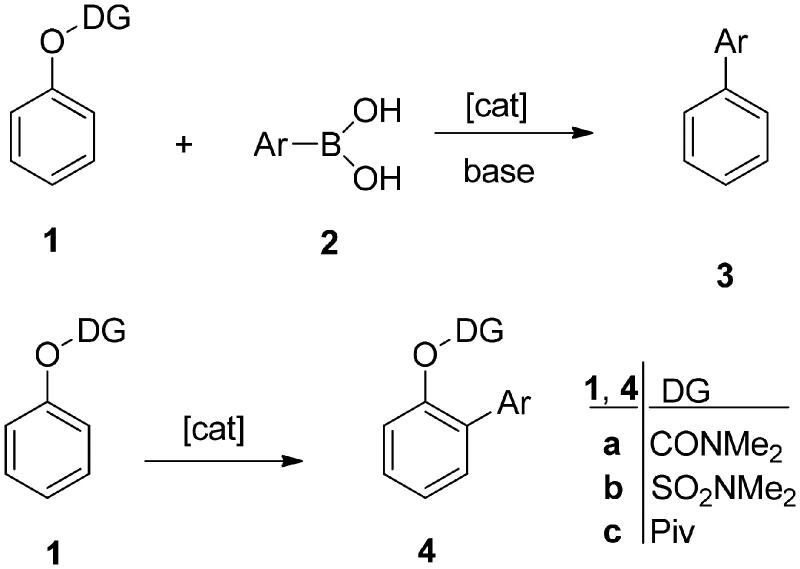
Formation of biaryl products by catalytic cross coupling or directed C–H activation.

At the beginning of the 2000s, transition metals and, in particular, palladium came into focus in regard to the development of carbon–carbon coupling reactions. In principle, palladium is one of the few metals, which has a unique ability to activate various organic compounds. Essentially, two molecules are brought close to each other via forming metal–carbon bonds. The two partners couple together through establishing a new carbon–carbon single bond^1^. The significance of palladium-catalysed C–C cross couplings was highlighted by the shared Nobel Prize in Chemistry in 2010. Richard F. Heck, Ei-ichi Negishi, and Akira Suzuki received the Prize for “palladium-catalysed cross couplings in organic synthesis”^2^. These cross-coupling reactions have found remarkable utility in the synthesis of natural products and biologically active compounds. A wide range of their applications in the pharmaceutical industry increased their value even more. These coupling reactions are catalysed by zerovalent palladium, utilising organohalides as electrophilic and organometallic compounds as nucleophilic partners. Suzuki coupling facilitates the synthesis of biaryl compounds by employing organoboron nucleophiles^3^. Recently, direct arylations have come in the focus of attention. Aromatic C–H activation allows the formation of biaryl derivatives by avoiding the use of an organometallic nucleophilic partner. Palladium-catalysed chelation-directed C–H activations of phenol derivatives utilise “directing groups (DGs)”, which facilitate and direct the substitution in a regioselective manner. Literature describes several nitrogen-containing DGs, such as *N*-heterocycles, amides, imines, and amines^4–8^. In order to broaden the applicability of chelation-directed C–H activations, other types of substrates have been applied as well. Phenol esters^9–13^ offer a good alternative, owing to their usability under mild conditions (Scheme 1). Carbamates, sulfamates, or pivalates have already been examined as DGs in Pd-catalysed *ortho*-arylation of phenols^4^^,^^10^. These DGs are readily prepared, robust, easily removable, and might show important biological properties on their own right^10^. It should be emphasised that these phenolic derivatives are generally stable under Pd-catalysed reaction conditions^10–13^. Beside the synthetic advantages of aryl carbamates, sulfamates, or pivalates in directed C–H activations, these phenol derivatives are popular coupling partners in cross-coupling reactions (Scheme 1)^9^^,^^14^. The oxygen-based electrophiles provide a greener alternative to commonly used organohalides, since the halide-containing waste is avoided. Concerning the hydrolytic properties of the *O*-acylated phenol derivatives, the robust pivalate esters appear to be the right choice. Pivalates are attractive candidates considering cost and they are useful in both academic and industrial applications^15^. The pronounced stability of carbamates and sulfamates allows their application in coupling reactions using heterocyclic boronic acids^9^. Boronic acids are beneficial coupling partners in Suzuki–Miyaura reactions, owing to their wide availability, low toxicity, high stability, and broad functional group tolerance. Ni- or Fe-catalysed C(sp^2^)–C(sp^2^) couplings of the mentioned phenol derivatives with boronic acids or aryl trifluoroborates are known in the literature^9^^,^^14^^,^^16^. The methodology based on Ni-catalysed coupling of halogen-free substrates described recently, applying non-toxic boronic acids in green solvents, might find application even in industry^9^.

**Scheme 2.**
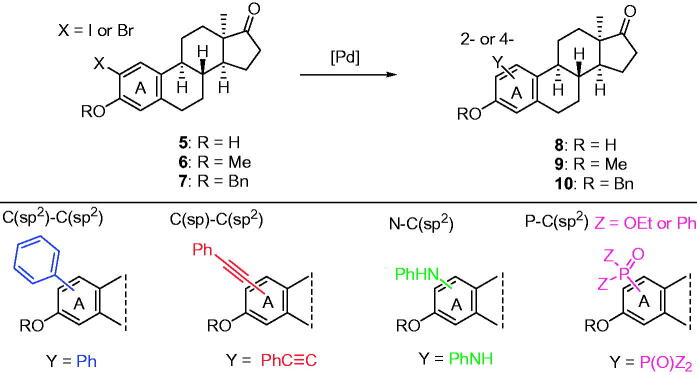
Pd-catalysed cross couplings of 13α-estrones as aryl halides.

We have recently described Pd-catalysed cross couplings at the C-2 or C-4 position of the 13α-estrane core (Scheme 2)^17–20^. Starting from steroidal aryl halides (**5**–**7**), microwave-assisted C–C, C–N, or C–P couplings were performed. The 2-(substituted 4-phenyl) moiety was attached to the A-ring of the steroid via C(sp^2^)–C(sp^2^) or C(sp^2^)–C(sp) coupling. Suzuki–Miyaura reactions were carried out using substituted phenylboronic acids as reagents leading to biphenyl derivatives (**8**–**10**, Y═Ph)^17^. The indirect introduction of a phenyl group was performed in Sonogashira reactions through a linear C≡C linker, furnishing phenylethynyl derivatives (**8**–**10**, Y═PhC≡C)^18^. The Buchwald–Hartwig aminations of the bromoarene substrates (**5**–**7**) with aniline derivatives led to phenylamino compounds (**8**–**10**, Y═PhNH)^19^. The Hirao couplings facilitated the functionalisation of the A-ring with substituents differing in size and polarity^20^. Steroidal phosphonates and tertiary phosphine oxide derivatives (**8**–**10**, Y═P(O)Z_2_) were synthesised. In addition to positions C-2 or C-4, the 3-OH group was also modified by synthesising 3-*O*-alkyl and 3-*O*-aralkyl derivatives. The majority of 13α-oestrone derivatives modified in the A-ring proved to be biologically active. Certain biphenyl derivatives displayed substantial antiproliferative action against human reproductive cancer cell lines^17^. The 3-hydroxy-2-phenylethynyl compounds exerted marked inhibitory action against 17β-hydroxysteroid dehydrogenase 1 enzyme (17β-HSD1), which catalyses the last step of oestradiol biosynthesis^18^. C-2 phosphonated derivatives proved to be dual organic anion-transporting polypeptide (OATP2B1) and 17β-HSD1 inhibitors^20^. OATP2B1 is a membrane transporter facilitating the cellular uptake of various endogenous compounds, drugs, and hormonal steroids^21–23^. Since increased uptake of hormones by OATP2B1 might lead to marked tumour proliferation, inhibition of the transporter might be a powerful antitumoural approach^24^^,^^25^. It should be underlined that the biological activity of 13α-oestrone derivatives modified at the A-ring greatly depends on the substitution pattern of the aromatic ring. 13α-Oestrone is readily available from natural oestrone, and this core-modification results in marked decrease in the oestrogenic effect^26^^,^^27^. The latter suggest that 13α-oestrone is a valuable starting material in the design of bioactive oestrone-based agents lacking oestrogenic side effects.

Encouraged by our recent results, here we report the synthesis of 13α-oestrone carbamates, sulfamates, and pivalates suitable for C–H activation and cross-coupling reactions. Our study included microwave-assisted, Pd-catalysed regioselective *ortho*-arylations of phenol esters. Phenylation at C-3 was performed via Ni-catalysed Suzuki–Miyaura coupling using phenylboronic acid. Evaluation of *in vitro* antiproliferative action of the newly synthesised compounds against five human reproductive cancer cell lines was also accomplished.

## Materials and methods

Chemical syntheses, characterisation data of the reported compounds, as well as experimental conditions of antiproliferative assays performed are described in the Supporting Information.

## Results and discussion

### Chemistry

**Scheme 3.**
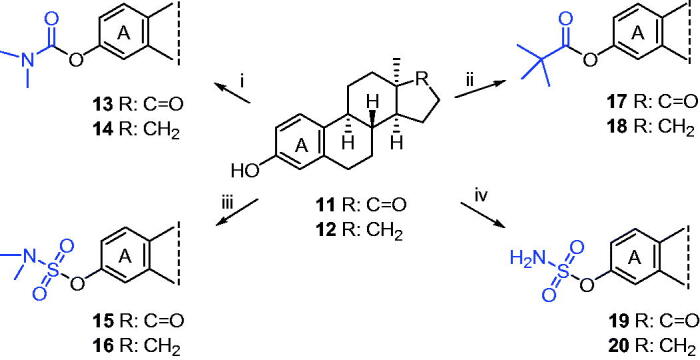
Syntheses of 13α-oestrone carbamates (**13**, **14**), pivalates (**17**, **18**), and sulfamates (**15**, **16**, **19**, **20**). Reagents and conditions: (i) *N*,*N*-dimethylcarbamoyl chloride (1.0 equiv.), NaH (1.3 equiv.), DMF, rt, 30 min; (ii) pivaloyl chloride (1.2 equiv.), DMAP (0.1 equiv.), NEt_3_ (1.2 equiv.), CH_2_Cl_2_, MW, 40 °C, 1 h; (iii) *N,N*-dimethylsulfamoyl chloride (1.0 equiv.), NaH (1.3 equiv.), toluene, MW, 100 °C, 30 min; (iv) sulfamoyl chloride (1.0 equiv.), NaH (1.3 equiv.), toluene, MW, 75 °C or 100 °C, 30 min.

Modification of the phenolic hydroxyl function with DGs was carried out starting from 13α-oestrone (**11**) or its 17-deoxy counterpart (**12**, Scheme 3). Reactions of the steroidal substrates with *N*,*N*-dimethylcarbamoyl chloride using sodium hydride as a base afforded the desired carbamate esters (**13**, **14**) in high yields. Pivalate and sulfamate derivatives (**15**–**20**) were synthesised in a microwave reactor. Esterifications of the substrates (**11** or **12**) using pivaloyl chloride as a reagent and triethylamine and (4-dimethylamino)pyridine as bases furnished the required pivalates (**17**, **18**) in excellent yields. Microwave irradiation of the starting compounds (**11** or **12**) with sodium hydride and sulfamoyl or *N,N*-dimethylsulfamoyl chloride yielded the corresponding sulfamates (**15**, **16**, **19**, **20**) in moderate to high yields.

There is information in the literature about the syntheses of 3-deoxy-3-phenyl derivatives of natural oestrone, starting from its trifluoromethanesulfonyl or triazinyl ester^28–31^. The cross-coupling reactions of the esters with boronic acid derivatives afforded the desired products. However, certain less common, but more robust phenol derivatives might also be utilised as substrates in cross-coupling reactions. Quasdorf et al. described Suzuki–Miyaura coupling reactions of phenol carbamates, sulfamates, and pivalates^32^^,^^33^. The activation of a rather inert aryl C–O bond was achieved via NiCl_2_(PCy_3_)_2_-promoted transformations. The couplings with boronic acid reagents were accomplished under heating in the temperature range of 110–150 °C in 20–24 h.

**Scheme 4.**
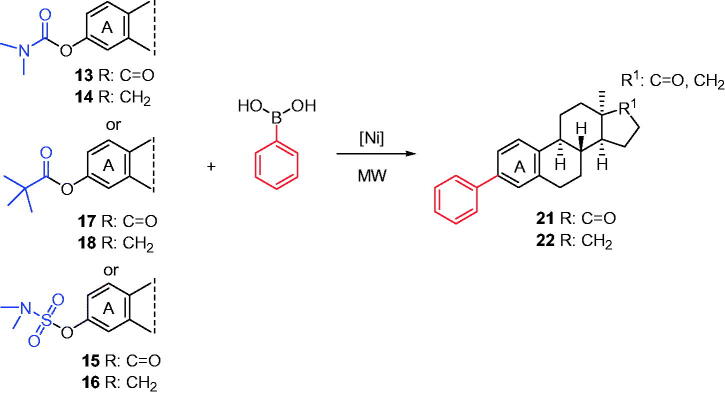
Syntheses of 3-deoxy-3-phenyl-13α-estrones (**21**, **22**). Reagents and conditions: from carbamates (**13** or **14**): NiCl_2_(PCy_3_)_2_ (10 mol%), phenylboronic acid (4 equiv.), K_3_PO_4_ (7.2 equiv.), toluene, MW, 130 °C, 1 h; from pivalates (**17** or **18**): NiCl_2_(PCy_3_)_2_ (10 mol%), phenylboronic acid (4 equiv.), K_3_PO_4_ (7.2 equiv.), MeCN, MW, 75 °C, 30 min; from sulfamates (**15** or **16**): NiCl_2_(PCy_3_)_2_ (10 mol%), phenylboronic acid (4 equiv.), K_3_PO_4_ (7.2 equiv.), toluene, MW, 130 °C, 1 h.

Here, we performed C(sp^2^)–C(sp^2^) couplings of 13α-oestrone esters according to the literature methodology^32^, but using microwave heating (Scheme 4). The Ni-catalysed couplings of steroidal substrates were carried out with phenylboronic acid as a reagent and K_3_PO_4_ as the base in various solvents. The Ni(II) precatalyst NiCl_2_(PCy_3_)_2_ is readily available and does not require glovebox handling. Conversions of carbamates (**13**, **14**) or sulfamates (**15**, **16**) were appreciably higher in toluene; however, reactions of pivalates (**17**, **18**) proceeded more effectively in acetonitrile. Reaction times varied from 30 to 60 min, depending on the nature of the DG. The most significant improvement in reaction time was observed in the case of pivalate (**17**, **18**) couplings. Owing to microwave irradiation, the reaction time could markedly be shortened, compared to those reported earlier applying other methodologies. It should be noted that 3-phenylation of 17-ketones took place with higher conversions than those of their 17-deoxy counterparts. The newly synthesised 3-deoxy-3-phenyl derivatives of 13α-oestrone (**21**, **22**) might be interesting from biological point of view. Biochemical investigations of the derivatives bearing a large, apolar moiety at C-3 might provide valuable structure–activity relationship. The removal of one or both oxygen-containing moieties (17-keto and/or 3-OH) might have great influence on the biological activity of the compounds.

Phenylations of natural or 13α-oestrone derivatives at C-2 were earlier carried out via Pd-catalysed cross-coupling reactions of steroidal aryl halides with boronic acid reagents^17^^,^^34^^,^^35^. However, this strategy requires multiple steps and prefunctionalisation of the reagents. An alternative methodology, based on C–H activation, might circumvent the inconveniences of cross-coupling reactions, such as halogenation of the substrates and the use of organometallic nucleophilic coupling partners. Regioselective C–H bond arylation of 3-carbamoylestrone with aryl iodides was described by Bedford et al.^36^ In this transformation, Pd(OAc)_2_ was used as a catalyst, AgOAc as a base in TFA solvent, and the mixture was stirred at 60 °C for 18 h. Arylation occurred at the C-2 *ortho*-position owing to the directing ability of the carbamate group. The removal of the DG was achieved in a subsequent step, by treatment with LiAlH_4_ followed by acidic hydrolysis. Note that not only 3-carbamoylestrone, but its 3-pivaloyl derivative also proved to be suitable for the regioselective arylation via C–H activation. Palladium-catalysed transformation of oestrone pivalate with the appropriate hypervalent iodonium salt gave the 2-tolyl derivative^37^. The reaction mixture was stirred at room temperature for 24 h.

**Scheme 5.**
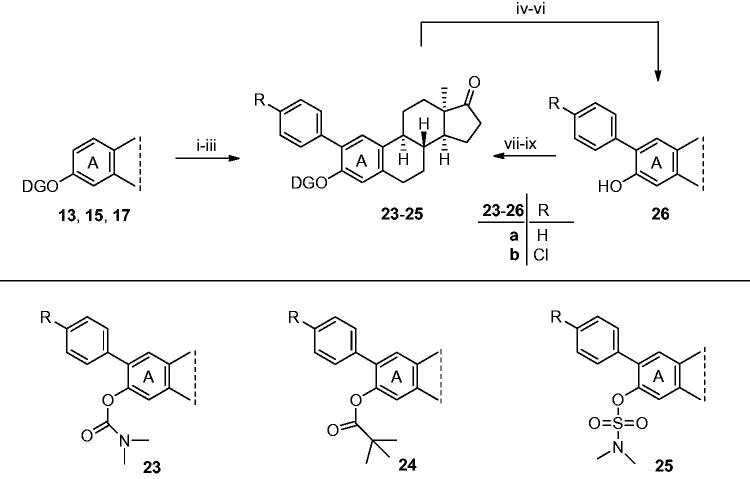
Syntheses of 2-(4-substituted phenyl)-13α-oestrone derivatives (**23a**,**b**–**25a**,**b**). Reagents and conditions: (i) carbamate **13**, Pd(OAc)_2_ (10 mol%), iodobenzene (4 equiv.) or 1-chloro-4-iodobenzene (4 equiv.), K_2_CO_3_ (2 equiv.), TFA, MW, 50 °C, 60 min; (ii) pivalate **17**, Pd(OAc)_2_ (10 mol%), iodobenzene (4 equiv.) or 1-chloro-4-iodobenzene (4 equiv.), K_2_CO_3_ (2 equiv.), TFA, MW, 50 °C, 60 min; (iii) sulfamate **15**, Pd(OAc)_2_ (10 mol%), iodobenzene (4 equiv.) or 1-chloro-4-iodobenzene (4 equiv.), K_2_CO_3_ (2 equiv.), TFA, MW, 50 °C, 60 min; (iv) 2-phenyl carbamate **23a**, TFA, MW, 150 °C, 30 min; (v) 2-phenyl pivalate **24a**, TFA, MW, 100 °C, 30 min; (vi) 2-phenyl sulfamate **25a**, TFA, MW, 150 °C, 30 min; (vii) *N*,*N*-dimethylcarbamoyl chloride (1.0 equiv.), NaH (1.3 equiv.), DMF, rt, 30 min; (viii) pivaloyl chloride (1.2 equiv.), DMAP (0.1 equiv.), NEt_3_ (1.2 equiv.), CH_2_Cl_2_, MW, 40 °C, 1 h; (ix) *N,N*-dimethylsulfamoyl chloride (1.0 equiv.), NaH (1.3 equiv.), toluene, MW, 100 °C, 30 min.

With these considerations in mind, here we intended to explore the applicability of the earlier elaborated C–H activation methodologies on the hormonally inactive 13α-oestrone, utilising microwave irradiation (Scheme 5). The newly synthesised 3-carbamoyl (**13**), -pivaloyl (**17**), or -sulfamoyl (**15**) 13α-oestrone derivatives were selected as substrates. The rational of this choice included both the DG ability of the mentioned 3-*O*-moieties and their important potential biological activities in their own right^10^^,^^38^^,^^39^. First, the transformation of 3-carbamoyl derivative **13** was carried out. As a first attempt, the combination of aryl iodide (4 equiv.) and silver acetate (2 equiv.) was used with Pd(OAc)_2_ (10 mol%) catalyst in TFA solvent. In earlier studies, this methodology proved to be suitable for *ortho*-arylation of anilides and *O*-carbamoylphenols^5^^,^^10^^,^^36^. The reaction of compound **13** with iodobenzene was performed under both conventional heating and in a microwave reactor at different temperatures. Microwave irradiation markedly improved the efficiency of the C–H activations, since conventional heating at 50 °C gave only moderate yields of the desired **23a** product. Microwave heating at 50 °C for 1 h, in turn, afforded the best results in site-selective 2-phenylation with both high product yields and high regio- and chemoselectivity. Formation of neither the 4-phenyl regioisomer nor 2,4-*bis* products was observed. The explanation of exclusive 2-selectivity might be the steric hindrance of the B-ring. Similar yields were obtained under the same reaction conditions (time and temperature) by replacing AgOAc (2 equiv.) with Cs_2_CO_3_ (2 equiv.) or K_2_CO_3_ (2 equiv.). Considering the price of the mentioned bases, K_2_CO_3_ is the best choice.

When the reaction mixture submitted to a 1-h irradiation at 50 °C was additionally exposed to a treatment at 150 °C for 1 h, complete removal of the DG occurred. Consequently, our present microwave-assisted methodology allows one-pot *ortho*-arylation and removal of the DG in a tandem reaction.

Next, we explored the reactions of 3-pivalate **17**. Substrate **17** was subjected to C–H activation by utilising the microwave-assisted methodology elaborated here. The desired 2-phenyl derivative (**24a**) was obtained only in moderate yield. The pivalate ester-directed approach seemed to be less efficient than that of the carbamate. According to the literature, in the reaction of pivalates with hypervalent iodonium salts, acyloxy-directed Pd(II)-insertion into the C–H bond of phenol derivatives might be promoted with the use of TFA as solvent^4^. The acid is capable of tuning the electrophilicity of the transition metal. However, in the case of 13α-oestrone pivalate **17**, phenylation with iodobenzene did not meet our expectations concerning product yield. If the reaction mixture was exposed to an additional microwave irradiation at 100 °C for 45 min, the pivaloyl DG could completely be removed. *N*,*N*-Dimethylsulfamoyl derivative **15** was also subjected to microwave-assisted C–H activations with Pd(OAc)_2_ as catalyst; however, no reaction occurred.

Next, we focussed on the synthesis of the 2-phenyl derivatives (**23**–**25**) via an alternative approach. The facile, one-pot, microwave-assisted methodology furnishing the 3-deprotected 2-phenyl derivative (**26a**) allowed the synthesis of the desired 13α-oestrone derivatives (**23a**–**25a**) in an indirect manner. 2-Phenyl-13α-oestrone (**26a**) was synthesised from its carbamate derivative (**23a**) according to the MW procedure described above, and a pivalate or a sulfamate DG was introduced in the next step. This two-step reaction route provided the 2-phenyl-3-protected derivatives (**24a**, **25a**) in high overall yields. Based on our recent publication, concerning the promising antiproliferative action of 2-(4-chlorophenyl)-13α-oestrone (**26b**)^17^, arylations with 1-chloro-4-iodobenzene were also performed. 2-(4-Chlorophenyl) derivatives (**23b**–**25b**) were synthesised via the two-step reaction pathway described above. C–H activations with Pd(OAc)_2_ as the catalyst, starting from carbamate **13** or pivalate **17** and 1-chloro-4-iodobenzene as the reagent, under the above-described microwave conditions, delivered the desired 2-(4-chlorophenyl) derivatives (**23b**–**25b**) in high yields. The presence of chlorine at *para* position relative to iodine seemed to be advantageous concerning the yields of the desired products. The indirect approach for the synthesis of 3-protected 2-substituted phenyl derivatives (**23b**–**25b**) included the introduction of the DGs onto the phenolic hydroxy function of compound **26b**, synthesised from carbamate **13** (Scheme 5, vi–viii).

### Pharmacology

The antiproliferative properties of the newly synthesised compounds (**13**–**25**) and their parent derivatives **11** or **12** were investigated *in vitro* on a panel of human adherent cancer cell lines representing the most relevant cancers of gynaecological origin. The panel included different breast (MCF-7 and MDA-MB-231), cervical (HeLa and SiHa), and ovarian (A2780^40^^,^^41^) cancer cell lines. While MCF-7 is an oestrogen receptor positive cell line, MDA-MB-231 is lacking receptors for oestrogens, progesterone, and HER2 growth factor^41–44^. HeLa and SiHa differ in their HPV status: they represent HPV-18 and HPV-16 positive cases, respectively^45^^,^^46^. The determination of cell growth inhibitory action of test compounds was performed by MTT assay^47^. The cancer selectivity of the most effective derivatives was additionally determined by the same method using NIH/3T3 mouse fibroblast cells.

We have recently reported the synthesis of potent antiproliferative core-modified oestrone derivatives obtained by inversion of the C-13 configuration or opening the D-ring^17^^,^^48–55^. Certain test compounds displayed submicromolar IC_50_ values, occasionally with high cell line selectivity. The 13α- and/or D-seco compounds possessed modifications mainly at positions C-2, C-4, and/or 3-OH. It was shown that the nature, size, and polarity of the introduced substituents greatly influence the cell growth inhibitory properties of the compounds. 3-Hydroxy derivatives proved to be generally less potent than their 3-ether counterparts. Introduction of a benzyl or benzyltriazolyl group onto 3-OH improved the antiproliferative properties substantially^51–55^. Having found functional groups responsible for the biological effect, we turned our attention to attach all crucial structural elements to the hormonally inactive 13α-oestrone core. As a result, certain derivatives were identified as dually acting agents, possessing both enzyme inhibitory and antiproliferative properties^20^^,^^55^. We demonstrated that a few of our potent anticancer compounds exert a direct effect on the tubulin-microtubule system by increasing the rate of tubulin polymerisation^50^^,^^52^^,^^55^. These compounds might give an important basis for the design of oestrone-based potent anticancer agents, lacking oestrogenic activity.

Concerning the modification of the phenolic hydroxy function of oestrone derivatives, important structure–activity relationships are described in the literature. Introduction of a sulfamoyl moiety onto 3-OH of oestrogens resulted in compounds possessing remarkable biological activities^56–59^. Their aryl *O*-sulfamate pharmacophore facilitated steroid sulfatase inhibitory activity, leading to anticancer effect. Nevertheless, certain oestrone sulfamates proved to be active against triple-negative breast cancer owing to their triple effect^60^. Beside their microtubule disruptor properties, they displayed proapoptotic and anti-angiogenic action^60^. Furthermore, carbamoylation of oestrogens at C-3-*O* afforded *N*-mustard carbamates as potent cytotoxic agents against prostatic adenocarcinoma^61^.

In addition to modifications at C-3, introduction of substituents onto C-2 of the estrane core led to potent anticancer agents of high value. 2-Halogenated and 2-phenylated derivatives were identified as efficient inhibitors against enzymes involved in the metabolism or biosynthesis of oestrogens^34^^,^^62^. It was highlighted that 2-phenyl derivatives display potent CYP1B1 inhibitory action^34^. CYP1B1 is responsible for the bioactivation of certain procarcinogens, thereby catalysing the synthesis of mutagenic compounds. The combination of a CYP1B1 inhibitor with an anticancer agent might be suitable for the treatment of drug-resistant cancers^63^^,^^64^. We recently described our results with respect to the antiproliferative action of 2- or 4-(substituted phenyl)-13α-estrones and their 3-benzyl ethers^17^. 2-(4-Chlorophenyl)-13α-oestrone was found to be the most potent compound with low micromolar cell growth inhibitory action against MCF-7 and HeLa cell lines. An important structure–activity relationship was found, since the 3-benzyl ether counterpart proved to be ineffective. A substantial distinction was observed between the two pairs of breast and cervical cancer cell lines, concerning the cell growth inhibitory action. The triple negative breast and the HPV-16 positive cervical cell lines seemed to be less sensitive to the test compound.

Taking into consideration of the above-mentioned promising pharmacological properties of natural and 13α-oestrone derivatives, here we combined the essential structural elements with the aim of getting more potent antiproliferative agents and important structure–activity relationship data. The key pharmacophores identified on the natural estrane core were transferred to the hormonally inactive 13α-estone basic compound. Before testing the newly designed derivatives, the cell growth inhibitory properties of the starting 13α-oestrone (**11**) and its 17-deoxy counterpart (**12**) were compared. As it can be seen in Table 1, compound **12** lacking the 17-keto group displayed stronger inhibitory actions than 13α-oestrone **11**. As a consequence, it seemed rational to test the influence of the modification at the C-3-*O* group on both 17-keto and 17-deoxy core structures on the antitumoural action. Carbamoylation leading to compounds **13** or **14** did not improve the cell growth inhibitory properties. However, introduction of a pivaloyl moiety seemed to lower the IC_50_ values of 17-deoxy derivative **18** (against MCF-7 and HeLa). The most potent compound group is represented by the 3-*O*-sulfamoyl derivatives. Sulfamates bearing NH_2_-function (**19**, **20**) generally exerted less potent action, than the *N*,*N*-dimethyl derivatives (**15**, **16**). The latter structural element, combined with a 17-keto group resulted in a highly potent derivative (**15**). Phenylations at C-3 via Suzuki–Miyaura coupling afforded 3-deoxy-3-phenyl derivatives (**21**, **22**), possessing weak antiproliferative action. These results indicate that introduction of a large, apolar moiety onto C-3 by the simultaneous removal of the oxygen-containing moiety is rather disadvantageous concerning the cell growth inhibitory action on the tested cell lines. Summarising the results obtained for C-3-*O*-modified 13α-oestrone derivatives, it can be emphasised, that the determined antiproliferative effect greatly depends on the nature of introduced moieties. This screening suggests that compound **15** is the most promising candidate for further evaluations.

**Table 1.** Antiproliferative properties of the synthesised compounds.

Comp. number	Conc. (μM)	Inhibition (%)±SEM (calculated IC_50_)^a^					
		MCF-7	MDA-MB-231	HeLa	SiHa	A2780	NIH-3T3
11	10	23.04 ± 1.25	–^b^	–	–	–	n.t.
	30	29.05 ± 2.75	–	23.38 ± 1.56	–	24.39 ± 2.24	
12	10	25.69 ± 2.26	18.34 ± 2.01	33.82 ± 0.92	–	–	–
	30	95.96 ± 0.54 (13.65)	96.18 ± 0.43 (14.17)	99.01 ± 0.97 (12.00)	96.04 ± 0.32 (15.80)	97.80 ± 0.85 (13.69)	97.05 ± 0.15 (15.29)
13	10	27.18 ± 1.67	–	23.78 ± 1.28	32.27 ± 1.03	21.99 ± 1.12	n.t.
	30	38.53 ± 1.26	–	66.10 ± 1.87 (17.26)	35.01 ± 0.94	45.81 ± 0.92	
14	10	–	–	–	–	–	n.t.
	30	26.60 ± 1.97	–	54.70 ± 1.35	22.26 ± 1.49	36.57 ± 1.99	
15	10	60.47 ± 2.62	15.48 ± 2.29	67.84 ± 0.86	58.35 ± 0.66	23.38 ± 1.50	23.98 ± 2.16
	30	82.67 ± 1.15 (5.28)	42.14 ± 1.23	69.78 ± 1.08 (6.67)	60.32 ± 1.09 (13.21)	40.16 ± 2.09	44.12 ± 2.35
16	10	62.39 ± 1.61	24.89 ± 1.46	56.34 ± 0.69	55.25 ± 1.27	24.89 ± 2.05	32.77 ± 0.61
	30	88.70 ± 1.44 (5.54)	57.66 ± 1.67 (24.15)	60.84 ± 1.50 (9.49)	58.80 ± 0.83 (13.07)	48.72 ± 2.53	47.71 ± 0.92
17	10	48.02 ± 0.67	–	49.48 ± 1.41	–	–	n.t.
	30	60.59 ± 0.77 (14.60)	36.93 ± 2.25	66.08 ± 0.92 (13.11)	52.15 ± 0.77	30.81 ± 0.67	
18	10	53.74 ± 0.44	32.70 ± 0.51	66.02 ± 1.43	49.83 ± 1.12	–	29.96 ± 0.99
	30	60.39 ± 0.87 (9.14)	35.72 ± 0.47	68.20 ± 1.16 (6.34)	50.91 ± 1.84	35.21 ± 1.76	33.69 ± 1.23
19	10	21.30 ± 0.31	–	46.89 ± 1.72	–	–	n.t.
	30	29.57 ± 1.32	44.55 ± 1.07	66.55 ± 1.20 (11.80)	–	46.78 ± 1.89	
20	10	33.48 ± 1.58	–	73.19 ± 2.04	–	–	–
	30	80.58 ± 1.39 (12.14)	35.91 ± 1.02	85.41 ± 0.96(6.90)	84.63 ± 1.23 (21.49)	83.48 ± 0.60 (16.86)	25.02 ± 2.20
21	10	22.97 ± 2.41	–	–	–	27.25 ± 1.20	n.t.
	30	42.59 ± 2.77	–	63.61 ± 2.00 (19.62)	20.84 ± 1.54	42.05 ± 1.04	
22	10	–	–	–	–	–	n.t.
	30	23.15 ± 0.77	–	21.20 ± 2.10	25.27 ± 0.57	–	
23a	10	23.48 ± 1.63	–	–	21.84 ± 1.76	33.53 ± 2.15	n.t.
	30	87.72 ± 0.70 (13.95)	67.95 ± 0.92 (20.83)	52.74 ± 2.13	48.65 ± 1.95	89.68 ± 0.54 (12.99)	
23b	10	20.53 ± 3.05	–	48.03 ± 1.44	20.00 ± 1.57	–	24.62 ± 1.15
	30	58.51 ± 2.17 (24.01)	21.22 ± 2.19	65.31 ± 1.49 (13.48)	47.84 ± 0.80	55.00 ± 0.96	42.70 ± 1.32
24a	10	25.85 ± 1.93	–	48.76 ± 1.50	–	20.22 ± 2.27	–
	30	77.96 ± 2.01 (11.80)	31.40 ± 2.86	58.91 ± 0.76 (15.20)	41.32 ± 2.08	55.19 ± 0.36 (25.54)	32.11 ± 2.70
24b	10	66.09 ± 1.84	–	55.99 ± 1.62	–	–	–
	30	79.06 ± 3.18 (7.16)	–	62.70 ± 1.49 (8.23)	37.25 ± 2.01	48.58 ± 1.14	–
25a	10	61.11 ± 2.36	29.40 ± 0.71	62.78 ± 0.47	48.91 ± 1.60	31.83 ± 1.45	21.04 ± 1.09
	30	75.83 ± 2.58 (6.72)	37.59 ± 0.70	69.39 ± 0.80 (7.53)	55.37 ± 0.77 (15.95)	41.57 ± 2.10	25.72 ± 2.72
25b	10	57.84 ± 1.56	35.66 ± 0.64	81.11 ± 0.67	78.53 ± 2.53	50.01 ± 1.04	34.27 ± 1.93
	30	81.64 ± 2.61 (6.36)	65.31 ± 1.94 (16.34)	95.45 ± 0.80 (2.28)	91.44 ± 0.94 (2.71)	76.55 ± 1.01 (10.60)	50.57 ± 1.14
Cisplatin	10	53.03 ± 2.29	–	42.61 ± 2.33	88.64 ± 0.50	83.57 ± 1.21	91.80 ± 0.39
	30	86.90 ± 1.22 (5.78)	71.47 ± 1.20 (19.13)	99.93 ± 0.26 (12.43)	90.18 ± 1.78 (7.84)	95.02 ± 0.28 (1.30)	93.68 ± 0.20 (2.70)

n.t.: not tested.

^a^ Mean value from two independent measurements with five parallel wells; standard deviation <20%.

b Inhibition values <20% are not presented.

Determination of the antiproliferative activities of the newly synthesised compounds was continued by testing 2-(substituted 4-phenyl)-13α-oestrone derivatives (**23a**,**b**–**25a**,**b**) bearing different DGs. In the carbamate compound group (**13**; **23a**,**b**), phenylations did not improve the cytostatic properties. 2-Phenyl pivalate (**24a**) displayed somewhat higher effect on MCF-7 cell line than that of its 2-H counterpart (**17**). However, the sulfamate compound set (**15**; **25a**,**b**) provides interesting correlation results. It is worth comparing the data obtained for the three 17-keto sulfamates, namely, compound **15** with no modification at C-2 and the two 2-phenyl derivatives (**25a**,**b**). There was no marked difference in the effects exerted on the MCF-7 cell line: each compound displayed high potency with IC_50_ values in the low micromolar range. HeLa seemed to be correspondingly sensitive to the sulfamates, but the cell growth inhibitory activity was improved by introducing the 4-chlorophenyl moiety onto C-2. The most significant improvement caused by 4-chlorophenylation was observed on SiHa and A2780 cell lines. The growth of HPV-16 positive cervical cells was substantially inhibited by compound **25b** in a very low micromolar range. To the best of our knowledge, this is the first 13α-oestrone derivative with such a high potency against SiHa described in the literature. This test compound, exerting outstanding activity against both HPV-18 and HPV-16 positive cervical cancers, might be of great importance in the design of anticancer agents targeting cervical carcinomas, since the majority of these carcinomas are caused by these two types of HPV. For invasive cervical cancer, HPV-16 is the most prevalent type (approximately 60%), HPV-18 is the second (15%), and HPV-45 is the third most common type^65^.

With the aim of getting preliminary results concerning the tumour selectivity of the detected action, certain test compounds were additionally tested against a mouse fibroblast cell line (NIH/3T3). 17-Deoxy-13α-oestrone **12** exerted the most potent antiproliferative action against the NIH/3T3 cell line with an IC_50_ value of 15.29 µM. However, the rest of the test compounds influenced the growth of the fibroblasts scarcely (less than 51% inhibition even at 30 μM). It can be stated, that the NIH/3T3 cells are more sensitive to reference agent cisplatin than to the 13α-oestrone derivatives tested.

## Conclusions

We introduced *N*- and/or *O*-containing DGs onto the phenolic 3-OH function of 13α-oestrone and its 17-deoxy counterpart. The resulting carbamate, pivalate, or sulfamate esters proved to be suitable for regioselective *ortho*-arylations via Pd-catalysed C–H activation. A mild and efficient microwave-assisted methodology was elaborated. Arylation of a carbamate or pivalate and the removal of the DG were achieved via a one-pot, tandem, microwave procedure. The newly synthesised phenol esters were suitable electrophilic substrates in microwave-induced, Ni-catalysed Suzuki–Miyaura couplings with phenylboronic acid as a nucleophilic reagent. Biphenyl derivatives formed by C(sp^2^)–C(sp^2^) couplings represent an interesting novel class of 13α-estrane derivatives lacking one or two oxygen-containing functionalities. The antitumoural properties of the newly synthesised 13α-oestrone derivatives were determined *in vitro* on five human cancer cell lines of gynaecological origin. Certain potent antiproliferative compounds were identified and important structure–activity relationships were established. Sulfamate derivatives seemed to be superior concerning their substantial antiproliferative potential. 2-(4-Chlorophenyl)-13α-oestrone sulfamate **25b** displayed outstanding growth inhibitory action against the two cervical cancer cell lines with different HPV-status. The presence of an *N*,*N*-dimethylsulfamate pharmacophore together with the 2-(4-chlorophenyl) moiety improved the antitumoural action. Considering that HPV-16 and HPV-18 play a causative role in the majority of cervical cancer cases, newly identified **25b** with its hormonally inactive core might be a promising candidate in the design of new anticancer agents acting selectively.

## Supplementary Material

Supplemental Material
