# Supplementary material for: Transition metal-catalysed A-ring C–H activations and C(sp2)–C(sp2) couplings in the 13α-oestrone series and in vitro evaluation of antiproliferative properties
Source: J Enzyme Inhib Med Chem. 2021 Mar 26;36(1):895–902. doi: 10.1080/14756366.2021.1900165 (PMC8008932; doi:10.1080/14756366.2021.1900165)
Supplement: Supplemental Material [file IENZ_A_1900165_SM2111.pdf]

Transition metal-catalyzed A-ring C–H activations and C(sp^2)–C(sp^2) couplings in the 13α-estrone series and *in vitro* evaluation of antiproliferative properties

Péter Traj,[1] Ali Hazhmat Abdolkhaliq,[2] Anett Németh,[1] Sámuel Trisztán Dajcs,[1] Ferenc Tömösi,[3] Tea Lanisnik-Rizner,[4] István Zupkó,[2] Erzsébet Mernyák[1]*

[1]Department of Organic Chemistry, University of Szeged, Dóm tér 8, H-6720 Szeged, Hungary

[2]Department of Pharmacodynamics and Biopharmacy, University of Szeged, Eötvös u. 6., H-6720 Szeged, Hungary

[3]Department of Medicinal Chemistry, University of Szeged, Dóm tér 8, H-6720 Szeged, Hungary

[4]Institute of Biochemistry, Faculty of Medicine, University of Ljubljana, 1000 Ljubljana, Slovenia

*Corresponding author. Tel.: +36 62 544277; fax: +36 62 544200 (E. Mernyák).

E-mail address: bobe@chem.u-szeged.hu (E. Mernyák).

Experimental procedures

1. Experimental

1.1. Chemistry

Melting points (Mp) were determined with a Kofler hot-stage apparatus and are uncorrected. Elemental analyses were performed with a Perkin-Elmer CHN analyzer model 2400. Thin-layer chromatography: silica gel 60 F254; layer thickness 0.2 mm (Merck); eluents (ss): 30% ethyl acetate/70% hexane, detection with I_2 or UV (365 nm) after spraying with 5% phosphomolybdic acid in 50% aqueous phosphoric acid and heating at 100–120 °C for 10 min. Flash chromatography: silica gel 60, 40–63 µm (Merck). Reactions under microwave irradiation were carried out with a CEM Corporation focused microwave system, Model Discover SP. The maximum power of irradiation was 200 W. ^{1}H NMR spectra were recorded in DMSO-d_6, $CDCl_3$ solution with a Bruker DRX-500 instrument at 500 MHz, with Me_4Si as internal standard. ^{13}C NMR spectra were recorded with the same instrument at 125 MHz under the same conditions. Mass spectrometry: full scan mass spectra of the compounds were acquired in the range of 50 to 1000 m/z with a Finnigan TSQ-7000 triple quadrupole mass spectrometer (Finnigan-MAT, San Jose, CA) equipped with a Finnigan electrospray ionization source. Analyses were performed in positive ion mode using flow injection mass spectrometry with a mobile phase of 50% aqueous acetonitrile containing 0.1 v/v% formic acid. The flow rate was 0.3 ml/min. Five µl aliquot of the samples were loaded into the flow. The ESI capillary was adjusted to 4.5 kV and N_2 was used as a nebulizer gas.

1.1.2. General procedure for the synthesis of carbamates (**13**, **14**, **23a**, **23b**)

13α-Estrone **11** (54 mg, 0.20 mmol) or 17-deoxy-13α-estrone **12** (51 mg, 0.2 mmol) or 2-phenyl-13α-estrone **26a** (69 mg, 0.20 mmol) or 2-(4-chlorophenyl)-13α-estrone **26b** (76 mg, 0.20 mmol) was dissolved in 1 mL DMF. The solution was cooled to 0 ºC in an ice bath. NaH (6.24 mg, 0.26 mmol, 60% oil dispersion) was added and the reaction mixture was stirred for 30 min, then dimethylcarbamoyl chloride (18.41 μL, 0.20 mmol) was added. The resulting suspension was allowed to warm to room temperature over 3 h. The reaction mixture was diluted with 25 mL EtOAc and washed with 3 x 15 mL water. The organic layer was dried over Na_2SO_4, concentrated *in vacuo* and the resulting residue was purified by column chromatography.

*1.2.1. 3-(N,N-Dimethylcarbamoyloxy)-13α-estra-1,3,5(10)-triene-17-one (**13**)*

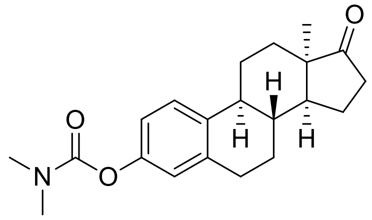

As described in Section 1.1.2, 13α-estrone **11** (54 mg, 0.2 mmol) was transformed. Purification by flash chromatography, using hexanes/EtOAc = 6:1 (v/v) as eluent afforded compound **13**, which was isolated as white crystals (63 mg, 92 %). Mp.: 134.7–135.4 °C; R_f: 0.15; M_r: 341.4; Anal. Calcd. for $C_{21}H_{27}NO_3$: C, 73.87; H, 7.97. Found: C, 73.96; H, 7.92; ^{1}H NMR (500 MHz, $CDCl_3$) δ ppm: 1.05 (s, 3H, 13-CH_3); 2.83 (m, 2H, 6-H_2); 2.99 and 3.07 (2xs, 2x3H, 2x*N*-CH_3); 6.81 (d, 1H, *J*= 2,5 Hz, 4-H); 6.86 (dd, 1H, *J*=8.5 Hz, *J*= 2.5 Hz, 2-H); 7.23 (d, 1H, *J*= 8.5 Hz, 1-H).^{13}C NMR ($CDCl_3$) δ ppm: 21.0 (CH_2); 25.1 (C-18); 28.2 (2xCH_2); 30.1 (CH_2); 32.1 (CH_2); 33.4 (CH_2); 36.4 and 36.6 (2x*N*-CH_3); 41.2 (CH); 41.6 (CH); 49.3 (CH); 50.1 (C-13); 119.0 (C-2); 121.6 (C-4); 126.7 (C-1); 136.6 (C-10); 138.0 (C-5); 149.3 (C); 155.2 (C); 221.6 (C-17). MS m/z (%) 342 (100, $[M+H]^+$).

*1.1.2.2. 3-(N,N-Dimethylcarbamoyloxy)-13α-estra-1,3,5(10)-triene (**14**)*

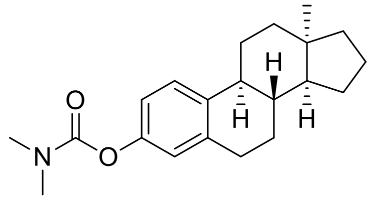

As described in Section 1.1.2, 17-deoxy-13α-estrone **12** (51 mg, 0.2 mmol) was transformed. Purification by flash chromatography, using hexanes/EtOAc = 9:1 (v/v) as eluent afforded compound **14**, which was isolated as white crystals (60 mg, 91 %). Mp.: 122.8–123.5 °C; R_f: 0.73; M_r: 327.5; Anal. Calcd. for $C_{21}H_{27}NO_2$: C, 77.02; H, 8.93. Found: C, 77.12; H, 8.87.

^{1}H NMR (500 MHz, $CDCl_3$) δ ppm: 0.99 (s, 3H, 13-CH_3); 2.83 (m, 2H, *J*= 8.7 Hz, *J*= 4.0, 2H, 6-H_2); 3.02 és 3.10 (2xs, 2x3H, 2x*N*-CH_3); 6.83 (d, 1H, *J*= 2.4 Hz, 4-H); 6.89 (dd, 1H, *J*=8.5 Hz, *J*= 2.4 Hz, 2-H); 7.30 (d, 1H, *J*= 8.5 Hz, 1-H).^{13}C NMR ($CDCl_3$) δ ppm: 20.9 (CH_2); 27.0 (CH_2); 27.9 (CH_2); 28.3(CH_2); 30.0 (C-18); 30.4 (CH_2); 33.7 (CH_2); 35.8 (CH_2); 36.4 és 36.7 (2x*N*-CH_3); 41.1 (CH); 41.9 (C-13); 42.5 (CH); 51.8 (CH); 118.8 (C-2); 121.5 (C-4); 126.8 (C-1); 137.7 (C-10); 138.4 (C-5); 149.2 (C); 155.2 (C). MS m/z (%) 328 (100, $[M+H]^+$).

*1.1.2.3. 3-(N,N-Dimethylcarbamoyloxy)-2-phenyl-13α-estra-1,3,5(10)-triene-17-one (**23a**)*

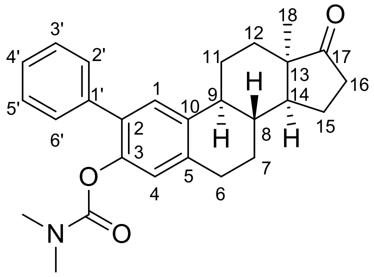

As described in Section 1.1.2, 2-phenyl-13α-estrone **26a** (69 mg, 0.20 mmol) was transformed. Purification by flash chromatography, using hexanes/EtOAc = 9:1 (v/v) as eluent afforded compound **23a**, which was isolated as white crystals (76 mg, 91 %). Mp.: 135.4–136.4 °C; R_f: 0.57. M_r: 417.5. Anal. Calcd. for $C_{27}H_{31}NO_3$: C, 77.67; H, 7.48. Found: C, 77.75; H, 7.41; ^{1}H NMR (500 MHz, DMSO-d$_6$) δ ppm: 0.98 (s, 3H, 13-CH_3); 2.76 and 2.86 (2xs, 2x3H, *N*-CH_3); 2.83 (m, 2H, 6-H_2); 6.86 (s, 1H, 4-H); 7.24 (s, 1H, 1-H); 7.31-7.42 (overlappping multiplets, 5H). ^{13}C NMR (DMSO-d$_6$) δ ppm: 20.4 (CH_2); 24.4 (C-18); 27.5 (CH_2); 27.8 (CH_2); 29.0 (CH_2); 31.5 (CH_2); 32.8 (CH_2); 35.8 and 36.1 (2x*N*-CH_3); 40.4 (CH); 40.8 (CH); 48.5 (CH); 49.3 (C-13); 123.0 (CH); 126.9 (CH); 127.5 (CH); 128.0 (2xCH); 128.5 (2xCH); 131.3 (C); 136.9 (C); 137.1 (C); 137.4 (C); 145,7 (C); 153.9 (C); 220,4 (C-17). MS m/z (%) 418 (100, $[M+H]^+$).

*1.1.2.4. 2-(4-Chlorophenyl)-3-(N,N-dimethylcarbamoyloxy)-13α-estra-1,3,5(10)-triene-17-one (**23b**)*

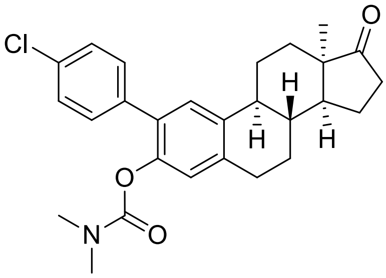

As described in Section 1.1.2, 2-(4-chlorophenyl)-13α-estrone **26b** (76 mg, 0.20 mmol) was transformed. Purification by flash chromatography, using hexanes/EtOAc = 6:1 (v/v) as eluent afforded compound **23b**, which was isolated as white crystals (84 mg, 93 %). Mp.: 152.2–153.2 °C; R_f: 0.38. M_r: 452.0. Anal. Calcd. for $C_{27}H_{30}ClNO_3$: C, 71.75; H, 6.69. Found: C, 71.81; H, 6.63.; ^{1}H NMR (500 MHz, $CDCl_3$) δ ppm: 1.06 (s, 3H, 13-CH_3); 2.84-2.90 (overlapping multiplets, 8H, 6-H_2 and 2x*N*-$(CH_3)_2$); 6.91 (s, 1H, 1-H); 7.31-7.35 (overlapping multiplets, 4H); ^{13}C NMR ($CDCl_3$) δ ppm:: 21.0 (CH_2); 25.1 (C-18); 28.2 (2x CH_2); 29.8 (CH_2); 32.0 (CH_2); 33.4 (CH_2); 36.3 and 36.7 (2x*N*-CH_3); 41.2 (CH); 41.6 (CH); 49.4 (CH); 50.1 (C-13);

123.1 (CH); 128.0 (C-1); 128.2 (2xCH); 130.4 (2xCH); 130.9 (C-10); 133.0 (C-5); 136.7 (C); 137.3 (C); 137.8 (C); 146.1 (C); 154.7 (C); 221.3 (C-17); MS m/z (%) 452 (100, $[M+H]^+$).

1.1.3. General procedure for the synthesis of pivalates (**17**, **18**, **24a**, **24b**)

The solution of 13α-estrone **11** (54 mg, 0.20 mmol) or 17-deoxy-13α-estrone **12** (51 mg, 0.2 mmol) or 2-phenyl-13α-estrone **26a** (69 mg, 0.20 mmol) or 2-(4-chlorophenyl)-13α-estrone **26b** (76 mg, 0.20 mmol) in dichloromethane (1 mL) was treated with triethylamine (33.50 μL, 0.240 mmol) and DMAP (2.50 mg, 0.020 mmol). Pivaloyl chloride (29.50 μL, 0.240 mmol, 1.2 equiv) was then added dropwise, over 5 min. The reaction mixture was heated in a CEM microwave reactor at 40 °C for 60 min under stirring in a 10 mL Pyrex pressure vessel (CEM, Part #: 908035) with silicone cap (CEM, Part #: 909210). A solution of aqueous $NaHSO_4$ (0.5 M, 25 mL) was added, and the layers were separated. The aqueous layer was extracted with CH_2Cl_2 (2 x 15 mL) and the combined organic layers were dried over Na_2SO_4. The solvent was removed under reduced pressure, the residue was purified by flash chromatography.

*1.1.3.1. 3-(Pivaloyloxy)-13α-estra-1,3,5(10)-triene-17-one (**17**)*

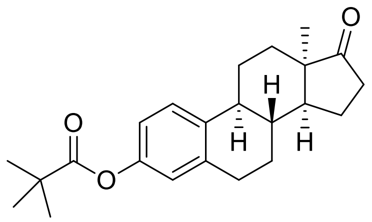

As described in Section 1.1.3, 13α-estrone **11** (54 mg, 0.20 mmol) was transformed. Purification by flash chromatography, using hexanes/EtOAc = 9:1 (v/v) as eluent afforded compound **17**, which was isolated as white crystals (66 mg, 93 %). Mp.: 142.6–143.6 °C; R_f: 0.69. M_r: 354.5. Anal. Calcd. for $C_{23}H_{30}O_3$: C, 77.93; H, 8.53. Found: C, 77.98; H, 8.47. ^{1}H NMR (500 MHz, $CDCl_3$) δ ppm: 1.06 (s, 3H, 13-CH_3); 1.34 (s, 9H, $C(CH_3)_3$); 2.84 (m, 2H, 6-H_2); 6.75 (d, 1H, *J*= 2.3 Hz, 4-H); 6.81 (dd, 1H, *J*= 8.5 Hz, *J*= 2.3 Hz, 2-H); 7.25 (d, 1H, *J*=8.5 Hz, 1-H). ^{13}C NMR ($CDCl_3$) δ ppm: 21.1 (CH_2); 25.1(C-18); 27.2 ($C(\underline{C}H_3)_3$; 28.2 (2xCH_2); 30.1 (CH_2); 32.1 (CH_2); 33.4 (CH_2); 39.0 ($\underline{C}(CH_3)_3$; 41.3 (CH); 41.7 (CH); 49.4 (CH); 50.1 (C-13); 118.7 (C-2); 121.2 (C-4); 126.8 (C-1); 137.1 (C-10); 138.2 (C-5); 149.0 (C-3); 177.2 (C=O), 221.3 (C-17). MS m/z (%): 355 (100, $[M+H]^+$).

*1.1.3.2. 3-(Pivaloyloxy)-13α-estra-1,3,5(10)-triene (**18**)*

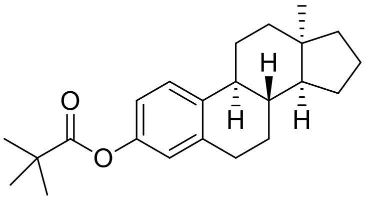

As described in Section 1.1.3, 17-deoxy-13α-estrone **12** (51 mg, 0.20 mmol) was transformed. Purification by flash chromatography, using hexanes/EtOAc = 9:1 (v/v) as eluent afforded compound **18**, which was isolated as white crystals (60 mg, 88 %). Mp.: 71.7–72.3°C; R_f: 0.88. M_r: 340.5. Anal. Calcd. for $C_{23}H_{32}O_2$: C, 81.13; H, 9.47. Found: C, 81.20; H, 9.41.

^{1}H NMR (500 MHz, $CDCl_3$) δ ppm: 0.97 (s, 3H, 13-CH_3), 1.34 (s, 9H, C(CH_3)$_3$); 2.81 (m, 2H, 6-H_2); 6.75 (d, 1H, *J*= 2,5 Hz, 4-H); 6.82 (dd, 1H, *J*= 8.5 Hz, *J*= 2,5 Hz, 2H); 7.29 (d, 1H, *J*= 8,5 Hz, 1-H). ^{13}C NMR ($CDCl_3$) δ ppm: 20.9 (CH_2); 27.0 (CH_2); 27.2 (C($\underline{C}H_3$)$_3$; 27.9 (CH_2); 28.2 (CH_2); 30.0 (C-18); 30.4 (CH_2); 33.7 (CH_2); 35.8 (CH_2); 39.0 ($\underline{C}$(CH_3)$_3$; 41.1(CH); 41.9 (C-13); 42.5 (CH); 51.8 (CH); 118.4 (C-2); 121.2 (C-4); 126.8 (C-1); 138.1 (C-10); 138.6 (C-5); 148.8 (C-3); 177.3 (C=O). MS m/z (%): 341 (100, $[M+H]^+$).

*1.1.3.3. 2-Phenyl-3-(pivaloyloxy)-13α-estra-1,3,5(10)-triene-17-one (**24a**)*

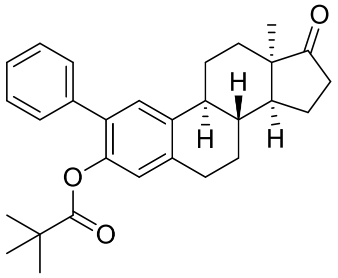

As described in Section 1.1.3, 2-phenyl-13α-estrone **26a** (69 mg, 0.20 mmol) was transformed. Purification by flash chromatography, using hexanes/EtOAc = 9:1 (v/v) as eluent afforded compound **24a**, which was isolated as white crystals (77 mg, 90 %). Mp.: 180.3–181.3 °C; R_f: 0.77. M_r: 430.6. Anal. Calcd. for $C_{29}H_{34}O_3$: C, 80.89; H, 7.96. Found: C, 80.94; H, 7.92. ^{1}H NMR (500 MHz, $CDCl_3$) δ ppm: 1.06 (s, 3H, 13-CH_3); 1.11 (s, 9H, C(CH_3)$_3$); 2.88 (m, 2H, 6-H_2); 6.78 (s, 1H, 4-H); 7.25 (s, 1H, 1-H); 7.29-7.36 (overlapping multiplets, 5H). ^{13}C NMR ($CDCl_3$) δ ppm:21.1 (CH_2); 25.1 (C-18); 27.0 (C($\underline{C}H_3$)$_3$); 28.2 (CH_2); 28.3 (CH_2); 29.8 (CH_2); 32.1 (CH_2); 33.5 (CH_2); 39.0 ($\underline{C}$(CH_3)$_3$); 41.3 (CH); 41.7 (CH); 49.5 (CH); 50.1 (C-13); 122.3 (C-4); 127.1 (C-1); 128.0 (2xCH); 128.4 (CH); 129.3 (2xCH); 132.6 (C); 137.4 (C); 137.5 (C); 138.0 (C); 146.0 (C); 176.9 (C=O); 221.2 (C-17). MS m/z (%) 431 (100, $[M+H]^+$).

*1.1.3.4. 2-(4-Chlorophenyl)-3-(pivaloyloxy)-13α-estra-1,3,5(10)-triene-17-one (**24b**)*

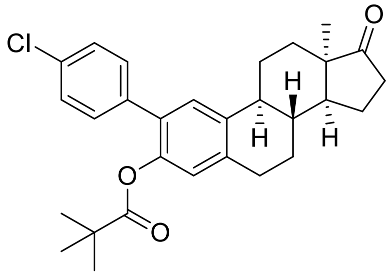

As described in Section 1.1.3, 2-(4-chlorophenyl)-13α-estrone **26b** (76 mg, 0.20 mmol) was transformed. Purification by flash chromatography, using hexanes/EtOAc = 13:1 (v/v) as eluent afforded compound **24b**, which was isolated as white crystals (85 mg, 91 %). Mp.: 126.8–127.5°C; R_f: 0.76. M_r: 465.0. Anal. Calcd. for $C_{29}H_{33}ClO_3$: C, 74.90; H, 7.15. Found: C, 74.98; H, 7.09. ^{1}H NMR (500 MHz, $CDCl_3$) δ ppm: 1.06 (s, 3H, 13-CH_3); 1.13 (s, 9H, $C(CH_3)_3$); 2.87 (m, 2H, 6-H_2); 6.78 (s, 1H, 4-H); 7.21 (s, 1H, 1-H); 7.27 (d, 2H, *J*=8.4 Hz); 7,33 (d, 2H, *J*=8.4 Hz). ^{13}C NMR ($CDCl_3$) δ ppm: 21.1 (CH_2); 25.1 (CH_3); 27.0 ($C(\underline{C}H_3)_3$); 28.2 (2xCH_2); 29.8 (CH_2); 32.0 (CH_2); 33.4 (CH_2); 39.0 ($\underline{C}(CH_3)_3$); 41.2 (CH); 41.7 (CH); 49.4 (CH); 50.1 (C-13); 122.5 (C-4); 128.2 (2xCH); 130.5 (2xCH); 131.3 (C); 133.2 (C); 136.4 (C); 137.7 (C); 137.9 (C); 145.8 (C); 177.0 (C=O); 221.3 (C-17). MS m/z (%) 465 (100, $[M+H]^+$).

1.1.4. General procedure for the synthesis of sulfamates (**15**, **16**, **19**, **20**, **25a**, **25b**)

A round bottom flask was charged with NaH (0.60 g, 15.12 mmol, 60% dispersion in oil). Then a solution of 13α-estrone **11** (54 mg, 0.20 mmol) or 17-deoxy-13α-estrone **12** (51 mg, 0.2 mmol) or 2-phenyl-13α-estrone **26a** (69 mg, 0.20 mmol) or 2-(4-chlorophenyl)-13α-estrone **26b** (76 mg, 0.20 mmol) in toluene (1 mL) or acetonitrile (1 mL) was added dropwise to the NaH. A solution of dimethylsulfamoyl chloride (43 μL, 0.400 mmol, 2 equiv) in toluene (1 mL) or acetonitrile (1 mL) was then added dropwise to the reaction vessel. The reaction mixture was heated in a CEM microwave reactor at 75 °C or 100 °C for 30 min under stirring in a 10 mL Pyrex pressure vessel (CEM, Part #: 908035) with silicone cap (CEM, Part #: 909210). The reaction was quenched with H_2O (5 mL), the mixture was extracted with ethyl acetate (3 x 15 mL). The combined organic layers were then washed with brine (15 mL), dried over Na_2SO_4, and concentrated under reduced pressure. The residue was purified by flash chromatography.

*1.1.4.1. 3-(Sulfamoyloxy)-13α-estra-1,3,5(10)-triene-17-one (**19**)*

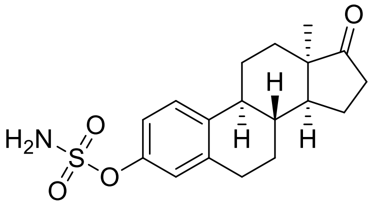

As described in Section 1.1.4, 13α-estrone **11** (54 mg, 0.20 mmol) was transformed in toluene at 100 °C. Purification by flash chromatography, using hexanes/EtOAc = 7:3 (v/v) as eluent afforded compound **19**, which was isolated as white crystals (61 mg, 88 %). Mp.: 202.4–203.1 °C; R_f: 0.17. M_r: 349.4. Anal. Calcd. for $C_{18}H_{23}NO_4S$: C, 61.87; H, 6.63. Found: C, 61.94; H, 6.57. ^{1}H NMR (500 MHz, DMSO-d_6) δ ppm: 0.98 (s, 3H, 13-CH_3); 2.80 (m, 2H, 6-H_2); 6.96 (d, 1H, *J*= 2.5 Hz, 4-H); 7.01 (dd, 1H, *J*=8.5 Hz, *J*= 2.5 Hz, 2-H); 7.33 (d, 1H, *J*= 8.5 Hz, 1-H); 7.85 (s, 2H, NH_2). ^{13}C NMR (DMSO-d_6) δ ppm: 20.3 (CH_2); 24.4 (C-18); 27.3 (CH_2); 27.7 (CH_2); 29.4 (CH_2); 31.5 (CH_2); 32.7 (CH_2); 40.3 (CH); 40.8 (CH); 48.4 (CH); 49.3 (C-13); 119.1 (C-2); 121.5 (C-4); 127.0 (C-1); 137.8 (C-10); 138.3 (C-5); 147.8 (C-3); 220.4 (C-17). MS m/z (%) 350 (100, $[M+H]^+$).

*1.1.4.2. 3-(Sulfamoyloxy)-13α-estra-1,3,5(10)-triene (**20**)*

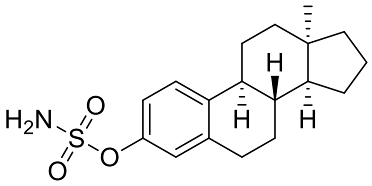

As described in Section 1.1.4, 13α-estrone **12** (51 mg, 0.20 mmol) was transformed in toluene at 100 °C. Purification by flash chromatography, using hexanes/EtOAc = 7:3 (v/v) as eluent afforded compound **20**, which was isolated as white crystals (50 mg, 75 %). Mp.: 122.6–123.3 °C; R_f: 0.64. M_r: 335.5. Anal. Calcd. for $C_{18}H_{25}NO_3S$: C, 64.45; H, 7.51. Found: C, 64.52; H, 7.46. ^{1}H NMR (500 MHz, $CDCl_3$) δ ppm: 0.98 (s, 3H, 13-CH_3); 2.83 (m, 2H, 6-H_2); 4.87 (s, 2H, NH_2); 7.02 (d, 1H, *J*= 2.5 Hz, 4-H); 7.08 (dd, 1H, *J*=8.5 Hz, *J*= 2.5 Hz, 2-H); 7.33 (d, 1H, *J*= 8.5 Hz, 1-H). ^{13}C NMR ($CDCl_3$) δ ppm: 20.9 (CH_2); 26.9 (CH_2); 28.0 (CH_2); 28.1 (CH_2); 30.0 (C-18); 30.4 (CH_2); 33.8 (CH_2); 35.8 (CH_2); 40.9 (CH); 41.9 (C); 42.6 (CH); 51.8 (CH); 118.9 (C-2); 121.7 (C-4); 127.4 (C-1); 139.4 (C-10); 140.2 (C-5); 147.8 (C-3). MS m/z (%) 359 (100, $[M+Na]^+$).

*1.1.4.3. 3-(N,N-Dimethylsulfamoyloxy)-13α-estra-1,3,5(10)-triene-17-one (**15**)*

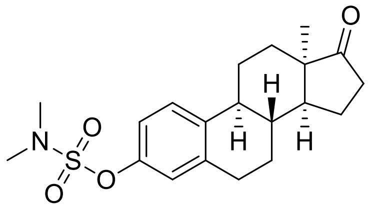

As described in Section 1.1.4, 13α-estrone **11** (54 mg, 0.20 mmol) was transformed in acetonitrile at 75 °C. Purification by flash chromatography, using hexanes/EtOAc = 6:1 (v/v) as eluent afforded compound **15**, which was isolated as white crystals (68 mg, 90 %). Mp.:169.2 –170.0 °C; R_f: 0.46. M_r: 377.5. Anal. Calcd. for $C_{20}H_{27}NO_4S$: C, 63.63; H, 7.21. Found: C, 63.70; H, 7.16. ^{1}H NMR (500 MHz, $CDCl_3$) δ ppm: 1.06 (s, 3H, 13-CH_3); 2.85 (m, 2H, 6-H_2); 2.96 (s, 6H, *N*-$(CH_3)_2$), 6.97–7.03 (overlapping multiplets, 2H, 2-H and 4-H) 7.25 (d, 1H, *J*= 7.8 Hz, 1-H). ^{13}C NMR ($CDCl_3$) δ ppm: 21.0 (CH_2); 25.1 (C-18); 28.1 (2xCH_2); 30.1 (CH_2); 30.3 (CH); 32.0 (CH_2); 33.3 (CH_2); 38.7 (CH); 41.2 and 41.7 (2x*N*-CH_3); 49.4 (CH); 50.1 (C-13); 118.8 (C-2); 121.4 (C-4); 127.1 (C-1); 138.4 (C-10); 138.7 (C-5); 148.1 (C-3); 221.0 (C-17). MS m/z (%) 378 (100, $[M+H]^+$).

*1.1.4.4. 3-(N,N-Dimethylsulfamoyloxy)-13α-estra-1,3,5(10)-triene (**16**)*

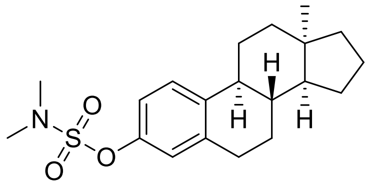

As described in Section 1.1.4, 17-deoxy-13α-estrone **12** (51 mg, 0.20 mmol) was transformed in toluene at 100 °C. Purification by flash chromatography, using hexanes/EtOAc = 6:1 (v/v) as eluent afforded compound **16**, which was isolated as white crystals (58 mg, 80 %). Mp.: 102.8–103.6°C; R_f: 0.79. M_r: 363.5. Anal. Calcd. for $C_{20}H_{29}NO_3S$: C, 66.08; H, 8.04. Found: C, 66.15; H, 8.00. ^{1}H NMR (500 MHz, $CDCl_3$) δ ppm: 0.97 (s, 3H, 13-CH_3); 2.83 (m, 2H, 6-H_2); 2.97 (s, 6H, *N*-$(CH_3)_2$), 6.98 (d, 1H, *J*= 2.5 Hz, 4-H); 7.03 (dd, 1H, *J*=8.5 Hz, *J*= 2.5 Hz, 2-H); 7.30 (d, 1H, *J*= 8.5 Hz, 1-H). ^{13}C NMR ($CDCl_3$) δ ppm: 21.0 (CH_2); 27.0 (CH_2); 28.0 (CH_2) ; 28.2 (CH_2); 30.0 (C-18); 30.4 (CH_2); 33.8 (CH_2); 35.8 (CH_2); 38.8 (2xCH); 41.0 and 42.5 (2x*N*-CH_3); 41.9 (C-13); 51,9 (CH); 118.6 (C-2); 121.4 (C-4); 127.2 (C-1); 139.2 (C-10); 139.5 (C-5); 148.0(C-3). MS m/z (%) 364 (100, $[M+H]^+$).

*1.1.4.5. 3-(N,N-Dimethylsulfamoyloxy)-2-phenyl-13α-estra-1,3,5(10)-triene-17-one (**25a**)*

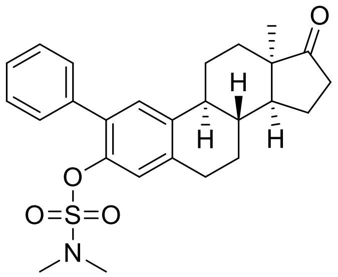

As described in Section 1.1.4, 2-phenyl-13α-estrone **26a** (69 mg, 0.20 mmol) was transformed in acetonitrile at 75 °C. Purification by flash chromatography, using hexanes/EtOAc = 7:1 (v/v) as eluent afforded compound **25a**, which was isolated as white crystals (83 mg, 92 %). Mp.: 216.5–217.2°C; R_f: 0.60. M_r: 453.6. Anal. Calcd. for $C_{26}H_{31}NO_4S$: C, 68.85; H, 6.89. Found: C, 68.92; H, 6.81; ^{1}H NMR (500 MHz, C_6D_6) δ ppm: 0.76 (s, 3H, 13-CH_3); 2.19 (s, 6H, 2x*N*-CH_3); 7.11 (t, 1H *J*= 7.3 Hz, 4'-H); 7.16 (s, 1H, 4-H); 7.19 (t, 2H, *J*= 7.3 Hz, 3'-H and 5'-H); 7.50 (d, 2H, *J*= 7.3 Hz, 2'-H and 6'-H); 7.58 (s, 1H, 1-H). ^{13}C NMR (C_6D_6) δ ppm: 20.6 (CH_2); 24.5 (C-18); 27.7 (CH_2); 27.9 (CH_2); 29.4 (CH_2); 31.8 (CH_2); 32.7 (CH_2); 37.4 (2x*N*-CH_3) 40.5 (CH); 41.4 (CH); 48.9 (CH); 49.3 (C-13); 122.5 (C-4); 127.1 (C-1); 128.0 (2xCH); 128.7 (C-4); 129.9 (2xCH); 132.3 (C); 138.0 (C); 138.1 (C); 138.2 (C); 145.5 (C); 218.1 (C-17); MS m/z (%) 454 (100, $[M+H]^+$).

*1.1.4.6. 2-(4-Chlorophenyl)-3-(N,N-dimethylsulfamoyloxy)-13α-estra-1,3,5(10)-triene-17-one (**25b**)*

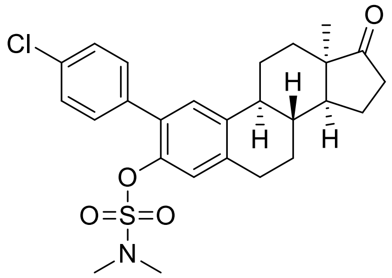

As described in Section 1.1.4, 2-(4-chlorophenyl)-13α-estrone **26b** (76 mg, 0.20 mmol) was transformed in acetonitrile at 75 °C. Purification by flash chromatography, using hexanes/EtOAc = 9:1 (v/v) as eluent afforded compound **25b**, which was isolated as white crystals (92 mg, 90 %). Mp.: 68.5–69.3 °C; R_f: 0.60. M_r: 488.0. Anal. Calcd. for $C_{26}H_{30}ClNO_4S$: C, 63.99; H, 6.20. Found: C, 64.07; H, 6.14. ^{1}H NMR (500 MHz $CDCl_3$) δ ppm: 1,07 (s, 3H, 13-CH_3); 2.60 (s, 6H, 2x*N*-CH_3); 2.90 (m, 2H, 6-H_2); 7.22 (s, 1H, 4-H); 7.25 (s, 1H, 1-H); 7.38 (s, 4H, 2'-,3'-5'- and 6'-H). ^{13}C NMR ($CDCl_3$) δ ppm: 21.1 (CH_2); 25.1 (C-18); 28.1 (2xCH_2); 29.9 (CH_2); 32.0 (CH_2); 33.4 (CH_2); 38.2 (2x*N*-CH_3); 41.2 (CH) 41.7 (CH); 49.4 (CH); 50.1 (C-13); 122.3 (C-4); 128.3 (2xCH); 128.6 (C-1); 131.0 (2xCH); 133.5 (C); 136.1 (C); 138.4 (2xC); 138.5 (CH); 145.0 (C); 221.0 (C-17); MS m/z (%) 488 (100, $[M+H]^+$).

1.1.5. General procedure for the Suzuki-Miyaura coupling of carbamates (**13**–**14**), pivalates (**17**–**18**) or sulfamates (**15**–**16**)

Aryl carbamate or pivalate or sulfamate (0.20 mmol), phenylboronic acid (98 mg, 0.80 mmol, 4 eq.), potassium phosphate (305 mg, 0.20 mmol, 7.2 eq.) and *bis*(tricyclohexylphosphine)-nickel(II) chloride (14 mg, 0,02 mmol, 10 mol%,) and solvent (3 mL) were added to a microwave vial. The mixture was irradiated at 130 °C or 75 °C. After cooling, ethyl acetate (mL) was added, and the mixture was washed with 25% aqueous ammonia (mL). The aqueous layer was extracted with ethyl acetate (3 x 15 mL). The combined organic layers were then washed with brine (15 mL), dried over Na_2SO_4, and concentrated under reduced pressure. The residue was purified by flash chromatography using hexanes/EtOAc = 9:1 (v/v) as eluent.

*1.1.5.1. 3-Phenyl-13α-estra-1,3,5(10)-triene-17-one (**21**)*

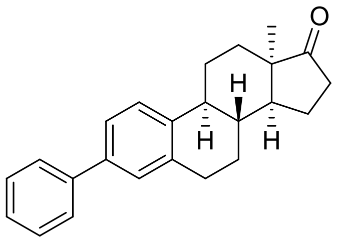

As described in Section 1.1.5, carbamate **13** (68 mg, 0.20 mmol) was reacted in toluene at 130 °C for 60 min. Compound **21** was isolated as white crystals (46 mg, 70 %).
As described in Section 1.1.5, pivalate **17** (71 mg, 0.20 mmol) was reacted in dioxane at 75 °C for 30 min. Compound **21** was isolated as white crystals (48 mg, 72 %).
As described in Section 1.1.5, sulfamate **15** (75 mg, 0.20 mmol) was reacted in DMF at 75 °C for 60 min. Compound **21** was isolated as white crystals (45 mg, 68 %).
Mp.: 151.3–152.0 °C; R_f: 0.44. Mr: 330.5. Anal. Calcd. for $C_{24}H_{26}O$: C, 87.23; H, 7.93. Found: C, 87.30; H, 7.87. ^{1}H NMR (500 MHz, $CDCl_3$) δ ppm: 1.08 (s, 3H, 18-H_3), 2.93 (m, 2H, 6-H_2), 7.30−7.43 (overlapping multiplets, 6H), 7.55−7.57 (m, 2H). ^{13}C NMR ($CDCl_3$) δ ppm: 21.1 (CH_2), 25.1 (C-18), 28.1 (CH_2), 28.3 (CH_2), 30.2 (CH_2), 32.1 (CH_2), 33.5 (CH_2), 41.3 (CH), 41.8 (CH), 49.4 (CH), 50.2 (C-13), 124.6 (C-2), 126.4 (C-4), 127.0 (3×CH), 127.5 (C-1), 128.7 (2×CH), 137.2 (C), 138.7 (C), 138.9 (C), 141.0 (C), 221.6 (C).

*1.1.5.2. 3-Phenyl-13α-estra-1,3,5(10)-triene (**21**)*

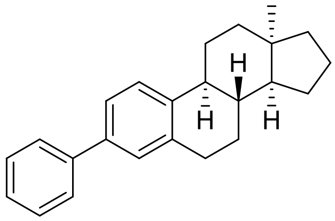

As described in Section 1.1.5, carbamate **14** (65 mg, 0.20 mmol) was reacted in toluene at 150 °C for 60 min. Compound **21** was isolated as white crystals (42 mg, 67 %).
As described in Section 1.1.5, pivalate **18** (68 mg, 0.20 mmol) was reacted in dioxane at 120 °C for 60 min. Compound **21** was isolated as white crystals (41 mg, 65 %).
As described in Section 1.1.5, sulfamate **16** (73 mg, 0.20 mmol) was reacted in DMF at 100 °C for 60 min. Compound **21** was isolated as white crystals (44 mg, 69 %).
Eluent: hexanes. Mp.: 100.3–101.2 °C; R_f: 0.94. M_r: 316.5. Anal. Calcd. for $C_{24}H_{28}$: C, 91.08; H, 8.92. Found: C, 91.12; H, 8.88; ^{1}H NMR (500 MHz, $CDCl_3$) δ ppm: 1.00 (s, 3H, 13-CH_3); 2.91 (m, 2H, 6-H_2); 7.31-7.34 (overlapping multiplets, 2H); 7.38-7.44 (overlapping multiplets, 4H; 7.59 (m, 2H). ^{13}C NMR ($CDCl_3$) δ ppm: 20.1 (CH_2); 26.9 (CH_2); 27.9 (CH_2); 28.4 (CH_2); 30.0 (C-18); 30.6 (CH_2); 33.7 (CH_2); 35.9 (CH_2); 41.2 (CH); 41.9 (C-13); 42.8 (CH); 51.9 (CH); 124.4 (CH); 126.4 (CH); 126.9 (CH); 127.0 (2xCH); 127.6 (CH); 128.6 (2xCH); 137.6 (C-10); 138.4 (C-5); 140.0 (C); 141.2 (C).

1.1.6. General procedure for the regioselective *ortho*-arylation of the carbamate **13** or pivalate **17**

The steroid (0.20 mmol), the aryl iodide (90 μL, 0.80 mmol, 4 eq.), the K_2CO_3 (55 mg, 0.40 mmol), the $Pd(OAc)_2$ (4.5 mg, 0.020 mmol) and TFA (1 ml) were added to a microwave vial. The reaction mixture was heated in a CEM microwave reactor at 50 °C for 60 min under stirring in a 10 mL Pyrex pressure vessel (CEM, Part #: 908035) with silicone cap (CEM, Part #: 909210). H_2O was added (5 ml) followed by $NaHCO_3$ (saturated solution, 10 ml) and the solution was extracted with 3 x 20 mL ethyl acetate. The combined organic layers were dried over Na_2SO_4, and the solvent removed under reduced pressure. The crude product was purified by column chromatography.

As described in Section 1.1.6, carbamate **13** (68 mg, 0.20 mmol) was transformed. Compound **23a** or **23b** was isolated as white crystals (75 mg, 90 % or 85 mg, 94 %, respectively).

As described in Section 1.1.6, pivalate **17** (71 mg, 0.20 mmol) was transformed. Compound **24a** or **24b** was isolated as white crystals (77 mg, 90 % or 86 mg, 92 %, respectively).

1.2. Determination of antiproliferative activities

The antiproliferative properties of the newly synthesized compounds (**3**, **6** and **11**–**16**) were determined on a panel of human adherent cancer cell lines of gynecological origin. MCF-7 and MDA-MB-231 were isolated from breast cancers differing in biochemical background, while A2780 cells were isolated from ovarian cancer. Hela and SiHa are cervical cancer cell lines positive for HPV-18 and HPV-16, respectively. The cancer selectivity of compounds was tested on the non-cancerous mouse embryo fibroblast cell line NIH/3T3. All cell lines were purchased from European Collection of Cell Cultures (ECCAC, Salisbury, UK) exception for Siha (American Tissue Culture Collection, Manassas, VA, USA). Cells were cultivated in minimal essential medium supplemented with 10% fetal bovine serum, 1% non-essential amino acids and an antibiotic–antimycotic mixture. All media and supplements were obtained from Lonza Group Ltd., Basel, Switzerland. Near-confluent cancer cells were seeded onto a 96-well microplate (5000 cells/well) and, after overnight standing, 200 µL new medium, containing the tested compounds at 10 and 30 µM, was added. After incubation for 72 h at 37 °C in humidified air containing 5% CO_2, the living cells were assayed by the addition of 20 µL of 5 mg/ml 3-(4,5-dimethylthiazol-2-yl)-2,5-diphenyltetrazolium bromide (MTT) solution. MTT was converted by intact mitochondrial reductase and precipitated as purple crystals during a 4-h contact period. The medium was next removed and the precipitated formazan crystals were dissolved in 100 µL of DMSO during a 60-min period of shaking at 37 °C.

Finally, the reduced MTT was assayed at 545 nm, using a microplate reader utilizing wells with untreated cells serving as control [S1]. In the case of the most active compounds (i.e. higher than 55% growth inhibition at 30 µM), the assays were repeated with a set of dilutions, sigmoidal dose–response curves were fitted to the determined data and the IC_{50} values (the concentration at which the extent of cell proliferation was half that of the untreated control) were calculated by means of GraphPad Prism 4.0 (GraphPad Software, San Diego, CA, USA). All in vitro experiments were carried out on two microplates with at least five parallel wells. Stock solutions of the tested substances (10 mM) were prepared in DMSO. The highest DMSO content of the medium (0.3%) did not have any substantial effect on cell proliferation. Cisplatin (Ebewe Pharma GmbH, Unterach, Austria) was used as positive control.

References:

[S1] T Mosmann. Rapid colorimetric assay for cellular growth and survival: application to proliferation and cytotoxicity assays. J Immunol Methods 1983;65:55–63.
